# Cost-Effectiveness and Resource Allocation (CERA) – directions for the future

**DOI:** 10.1186/1478-7547-7-14

**Published:** 2009-07-23

**Authors:** Rob Baltussen, Arnab Acharya, Kathryn Antioch, Dan Chisholm, Richard Grieve, Joses Kirigia, Tessa Tan Torres-Edejer, Damian G Walker, David Evans

**Affiliations:** 1 Department of Community and Primary Care, Radboud University Nijmegen Medical Center, Nijmegen, the Netherlands; 2 Health Policy Unit, London School of Hygiene and Tropical Medicine, London, UK; 3 Department of Epidemiology and Preventive Medicine, Monash University, Melbourne, Australia; 4 Department of Health Systems Financing, World Health Organization, Geneva, Switzerland; 5 Health Services Research Unit, London School of Hygiene and Tropical Medicine, London, UK; 6 Regional Advisor for Health Economics, World Health Organization – Regional Office for Africa, The Democratic Republic of the Congo; 7 Department of International health, Johns Hopkins Bloomberg School of Public Health, Baltimore, USA

## Abstract

The journal *Cost-Effectiveness and Resource Allocation *(CERA) is now in its seventh year, and is an excellent example of how open access publishing can improve dissemination. Now the journal is through its infancy, it is time to reflect on its orientation and to define the strategy for the years to come. Firstly, the journal will pay particular attention to stimulating and publishing studies originating from low- and middle-income countries. Second, CERA will continue to solicit contributions originating from high-income countries, but with the caveat that such studies should be of interest to the broad international readership of the journal. Third, the journal encourages submissions on methodological work from any setting, that is generalisable between low-, middle-, and high income countries. Fourth, CERA recognizes the development of national health accounts and expenditure tracking as a first step to improved resource allocation, and solicit manuscripts of this nature. Finally, CERA recognizes that cost and cost-effectiveness analysis alone may not provide sufficient information to decision makers to guide their choices on the allocation of resources, and therefore encourages submission of studies that advance the broader field of priority-setting.

## Editorial

Established in 2003, *Cost-Effectiveness and Resource Allocation *(CERA) is now in its seventh year. In this period, it has published 93 papers on various aspects of cost-effectiveness analysis, including conceptual or methodological work, economic evaluations, and policy analysis related to resource allocation at a national or international level. CERA is an Open Access online journal. The importance of this form of dissemination is well-recognized by research funders, such as the Medical Research Council and the Wellcome Trust, who insist that findings from research they fund is published in open access journals. CERA is an excellent example of how open access publishing can improve dissemination as illustrated by the large number of accesses to its articles: the 10 most popular articles have been accessed more than 8,000 times each, with the top-three accessed more than 21,000 times each. [1-3]

Now the journal is through its infancy, it is time to reflect on its orientation and to define the strategy for the years to come. This allows the journal to anticipate trends in cost-effectiveness and resource allocation in health, and to be an important journal in its field.

Firstly, the journal will pay particular attention to stimulating and publishing studies originating from low- and middle-income countries. There will never be sufficient resources available to allow all possible means of improving health to be provided to all people who might benefit from them. Rigorous comparisons of the relative health improvements from alternative uses of scarce resources are critical for informed decision-making. While this is true in any setting, the resource constraints are much more severe in low- and middle-income countries. Of all peer-reviewed articles on cost-effectiveness analysis published by journals in 2007, only 7% were set in developing nations and just eight were in Africa [4]. *CERA *aims to bridge this gap and be a home for this type of information. As part of its new approach to stimulating studies from the developing world, CERA offers fee-waivers for submissions originating from low-income countries.

Second, CERA will continue to solicit contributions originating from high-income countries, but with the caveat that such studies should be of interest to the broad international readership of the journal. In the past few years, the journal has received an increasing number of pharmacoeconomic submissions relating to specific interventions that focus on small patient numbers, mainly in high-income countries. We invite researchers in high-income countries to submit empirical work that has strong relevance *across *different settings (high, middle or low income). Where the focus is on the developed world, we request authors to explain why the results might be of more general relevance as well.

Third, we encourage methodological work from any setting, that is generalisable between low-, middle-, and high income countries. An example is the work by Mitton and Donaldson, on the principles, practice and challenges in health care priority setting. Another is the article by Bachmann and colleagues from South-Africa on the development of methods for analyzing cost effectiveness data from cluster randomized trials [5].

Fourth, CERA recognizes the development of national health accounts and expenditure tracking as a first step to improved resource allocation. We thereby solicit manuscripts that document the development or application of methodological advances in health accounting and resource tracking.

Finally, CERA recognizes that cost and cost-effectiveness analysis alone may not provide sufficient information to decision makers to guide their choices on the allocation of resources, and the implementation of interventions. Additional evidence on other relevant criteria, such as the budget impact of an intervention, whether an intervention targets disadvantaged populations, or the strength of evidence of its effectiveness is typically required. Additional challenges exist in addressing the many managerial questions decision-makers may have regarding the implementation of complex interventions. These questions can be considered to be part of the broader priority-setting process. CERA therefore encourages submission of studies that advance the broader field of priority-setting, in terms of methodological or conceptual contributions, as well as empirical case studies.

To help in this process, Richard Grieve and Kathryn Antioch have agreed to serve as Associate Editors. Richard Grieve has a particular interest in methods, while Kathryn Antioch will focus largely on studies from the developed world.

## Competing interests

The authors declare that they have no competing interests.

## Authors' contributions

All authors contributed to writing the manuscript, and all authors read and approved the final manuscript.
